# Tectorigenin Inhibits Glycolysis-induced Cell Growth and Proliferation by Modulating LncRNA CCAT2/miR-145 Pathway in Colorectal Cancer

**DOI:** 10.2174/0115680096274757231219072003

**Published:** 2024-01-11

**Authors:** Ying Xing, Bofan Lin, Baoxinzi Liu, Jie Shao, Zhichao Jin

**Affiliations:** 1 Department of Oncology, Affiliated Hospital of Nanjing University of Chinese Medicine, Nanjing, China;; 2 Jiangsu Province Hospital of Chinese Medicine, Nanjing, China

**Keywords:** Tectorigenin, glycolysis, colorectal cancer, lncRNA CCAT2, microRNA-145, cell counting kit-8 (CCK-8)

## Abstract

**Background:**

Colorectal cancer (CRC) places a heavy burden on global health. Tectorigenin (Tec) is a type of flavonoid-based compound obtained from the Chinese medical herb Leopard Lily Rhizome. It was found to exhibit remarkable anti-tumor properties in previous studies. However, the effect and molecular mechanisms of Tec in colorectal cancer have not been reported.

**Objective:**

The objective of this study was to explore the action of Tec in proliferation and glycolysis in CRC and the potential mechanism with regard to the long non-coding RNA (lncRNA) CCAT2/micro RNA-145(miR-145) pathway *in vitro* and *in vivo*.

**Methods:**

The anti-tumor effect of Tec in CRC was examined in cell and animal studies, applying Cell Counting Kit-8 (CCK-8) assay as well as xenograft model experiments. Assay kits were utilized to detect glucose consumption and lactate production in the supernatant of cells and animal serum. The expression of the glycolysis-related proteins was assessed by Western Blotting, and levels of lncRNA CCAT2 and miR-145 in CRC tissue specimens and cells were assessed by real-time quantitative PCR (RT-qPCR).

**Results:**

Tec significantly suppressed cell glycolysis and proliferative rate in CRC cells. It could decrease lncRNA CCAT2 in CRC cells but increase the expression of miR-145. LncRNA CCAT2 overexpression or inhibition of miR-145 could abolish the inhibitive effects of Tec on the proliferation and glycolysis of CRC cells. The miR-145 mimic rescued the increased cell viability and glycolysis levels caused by lncRNA CCAT2 overexpression. Tec significantly inhibited the growth and glycolysis of CRC xenograft tumor. The expression of lncRNA CCAT2 decreased while the expression of miR-145 increased after Tec treatment *in vivo*.

**Conclusion:**

Tec can inhibit the proliferation and glycolysis of CRC cells through the lncRNA CCAT2/miR-145 axis. Altogether, the potential targets discovered in this research are of great significance for CRC treatment and new drug development.

## 1 INTRODUCTION

Colorectal cancer (CRC), as a global health burden, is one of the most common cancers with the third highest incidence and the second mortality all over the world [1, 2]. Following deep research on cancer and the improvement of modern medicine, novel chemotherapy drugs and targeted therapies have been invented and come into clinical practice, relieving the pain of patients and prolonging their lives [3]. Meanwhile, the rapid development of drug preparation technology, such as nano-drug delivery systems, has brought more strategies and prospects for treating cancer [4, 5].

However, the benefits of systemic treatment remain limited due to issues, such as resistance to chemotherapy drugs or serious side effects [6, 7]. Hence, it is urgently required to achieve a breakthrough in the medical treatment of CRC.

Reprogramming energy metabolism, regulated by genetic changes and the tumor microenvironment, is regarded as an emerging hallmark of cancer [8], and aerobic glycolysis is the most frequently mentioned way of metabolic reprogramming. Even in the presence of oxygen, energy is produced by processing glucose into lactate in the cytoplasm of cancer cells, while mitochondrial oxidative phosphorylation for glucose catabolism occurs in normal cells [9]. A well-acknowledged fact about glycolysis in tumor cells is that tumor aerobic glycolysis is a critical contributor to malignant transformation, tumor proliferation, and distant metastasis [10-12]. Thus, it is one of the new therapeutic strategies for CRC by intervening in the aerobic glycolysis pathway to inhibit the growth and proliferation of tumor cells.

MicroRNAs (miRNAs) are endogenous non-coding RNAs consisting of about 18-25 nucleotides, which induce mRNA degradation or regulate the expression of target genes by binding to the target mRNAs [13]. As previously elucidated, miR-145, one of the most widely investigated miRNAs currently in cancer research, plays an essential role in regulating aerobic glycolysis in cancers. For example, miR-145 functioned as an inhibitor of the c-Myc/PTBP1/PKMs axis and down-regulate the expression of M2-type pyruvate kinase (PKM2), a key rate-limiting enzyme in aerobic glycolysis, to inhibit aerobic glycolysis in bladder cancer cells [14]. Zhang *et al.* also reported that a feedback loop between miR-145 and DNA methyltransferase (DNMT)3A is a potent target for the Warburg effect in ovarian cancer treatment [15]. LncRNAs have been disclosed to function as competing endogenous RNAs (ceRNAs), which regulate miRNA target gene expression by competitively binding to miRNA response elements (MREs). It was revealed that lncRNA colon cancer-associated transcript 2 (CCAT2) selectively blocks miR-145 maturation by inhibiting pre-miR-145 export to the cytoplasm [16]. However, the role of lncRNA CCAT2-miR-145 crosstalk in the glycolysis of CRC cells requires further investigation.

Chinese herbal medicine comprises multiple bioactive ingredients and provides adequate resources for new anticancer drugs [17]. Tectorigenin (Tec), a natural constituent isolated from leopard lily (Belamcanda chinensis L.) rhizome, has been reported to exert antitumor pharmacological effects in a few cancer types, including osteosarcoma [18], lung carcinoma [19], and hepatocellular carcinoma [20]. Nevertheless, the specific pharmacological action and underlying mechanisms of Tec in human colorectal cancer are largely unknown. In our studies, we investigated the impact of Tec on colorectal cancer cells HCT116 and explored whether Tec could exert inhibition of proliferation and glycolysis *via* the lncRNA CCAT2/miR-145 axis *in vitro* and *in vivo*.

## 2 MATERIALS AND METHODS

### 2.1 Cell Culture

HCT116, as a human colon cancer cell line, was purchased from the Cell Bank of Type Culture Collection of the Chinese Academy of Sciences (Shanghai, China). The cells were maintained in RPMI-1640 medium supplemented with 10% fetal bovine serum (FBS; Gibco, Thermo Fisher Scientific, Inc., Waltham, MA, USA) and 1% penicillin/streptomycin (Gibco) at 37°C with 5% CO_2_.

### 2.2 Reagents

Tectorigenin (HPLC≥98%) was obtained from Yuanye (Shanghai, China), and 5-fluorouracil (99.97%) was purchased from Selleck Chemicals (Houston, USA).

### 2.3 Cell Viability Assay

Cell viability was detected using the Cell Counting Kit-8 (CCK-8) assay. After the cancer cells were treated with drugs for 24 h in a 96-well plate, 10 μl CCK-8 solution was added to each well. The absorbance was read at 450 nm when the plate was incubated at 37°C for 1 h.

### 2.4 RNA Extraction and reAl-time Quantitative PCR (RT-qPCR)

Total RNA was isolated from cultured tissues or cells with FastPure Cell/Tissue Total RNA Isolation Kit (Vazyme, Nanjing) and reverse transcribed into cDNA using the HiScript III Reverse Transcriptase (Vazyme, Nanjing). The PCR reaction system was prepared using a Universal SYBR PCR Master Mix kit (Vazyme, China) according to the instructions. The 2^−∆∆Ct^ methods were applied to calculate the relative expression level of target genes. The primers were listed as follows: CCAT2: (5’-AGACAGTGCCAGCCAACC-3’, 5’-TGCCAAACCCTTCCCTTA-3’); PVT1: (5’- CCTGG-TGAAGCATCTGATGCACG-3’, 5’-GCCAGGCTTTGTG-GCACACGC-3’); miR-145: (5’-GTCCAGTTTTCCCAG-GAATCCC-3’, 5’-GTGCAGGGTCCGAGGT-3’); U6: (5’-GGCAGCACATATACTAAAATTG-3’, 5’-GGAACGCTT-CACGAATTTGCG-3’); GAPDH: (5’-CGGAGTCAACG-GATTTGGTCGTAT-3’, 5’-ACCCTTCTCCATGGTGGTG-AAGAC-3’).

### 2.5 Assays of Glucose Consumption and Lactate Production

The levels of glucose consumption and lactate production in the supernatant of cells and serum were detected using commercial assay kits (Sigma-Aldrich, USA) according to the manufacturer’s protocol.

### 2.6 Western Blotting Analysis

The total protein of cells or tissues was extracted by Radio Immunoprecipitation Assay (RIPA) lysis buffer (Beyotime Biotechnology, Shanghai) mixed with phenylmethanesulfonyl fluoride (PMSF) in a ratio of 100:1. The supernatant containing protein was collected and protein concentration was determined by a BCA assay kit (Beyotime Biotechnology, Shanghai). The whole protein lysate (20 μg) was separated by SDS-PAGE and transferred to PVDF membranes (Millipore, USA). Having been blocked in 5% skimmed milk at room temperature for 1 hour, the membranes were incubated with primary antibodies at 4°C overnight. Then, secondary antibodies were used to incubate the membranes at room temperature for 1 hour. Finally, the protein bands were imaged using a Bio-rad ChemiDoc XRS+ (Berkeley, USA).

### 2.7 Construction of Vectors and Cell Transfection

LncRNA CCAT2 overexpressed vector, miR-145 inhibitor/mimics, and their corresponding negative controls (NCs) were provided by Bioengineering Company (Shanghai, China). Lipofectamine 2000 was used to transfect plasmids into cells according to the manufacturer’s instructions (Invitrogen, USA). Expression of lncRNA CCAT2 and miR-145 was measured after 24 h transfection by RT-qPCR.

### 2.8 Tumor Xenograft Model *In vivo*

All animal experiments were approved by the Experimental Animal Ethics Committee of the Affiliated Hospital of Nanjing University of Chinese Medicine. To establish the cancer xenograft model, 0.2 ml HCT116 cell suspension was subcutaneously injected into the right underarm of 6-week-old athymic BALB/c nude mice (2 × 10^6^ /mouse). Seven days later, mice were blindly randomized into five groups: Control, Low-dose Tec, Median-dose Tec, High-dose Tec, and 5-Fu. Mice in the 5-Fu group were intraperitoneally injected with 0.2 ml 5-Fu (25 mg/kg) once every other day for 2 weeks, and mice in Tec groups were daily injected with 0.2 ml Tec (25 mg/kg, 50 mg/kg, and 75 mg/kg for low, median, and high doses, respectively) for 2 weeks. The mice in the control group were intraperitoneally injected with 200 μl saline solution every day for 2 weeks. Changes in the body weight and tumor volume of the animals were recorded during the experiment every week after the first drug treatment. Until the 35^th^ day, mice were sacrificed, and the tumors were collected.

### 2.9 Statistical Analysis

Half maximal inhibitory concentration (IC_50_) of Tec using the cell viability assay, statistical analyses, and graphing was determined using GraphPad Prism 9.0 software. Statistical analyses were performed using Student’s t-tests (unpaired, two-tailed) or the one-way analysis of variance (ANOVA) (followed by Tukey’s posthoc tests). Data in our study were represented as mean ± SD. P < 0.05 was regarded as statistically significant.

## 3 RESULTS

### 3.1 Tec Suppressed the Proliferation and Glycolysis of Colorectal Cancer Cells

To determine the anti-tumor effects of Tec on colorectal cancer cells and select the appropriate dose concentration for subsequent experiments, we first investigated changes in cell viability *via* CCK-8 assay. As shown in Fig. (**1A**), Tec treatment lasting for 24h reduced viable HCT116 cells in a concentration-dependent manner, with an IC_50_ of 141.0 μM and 140 μM Tec was chosen for the subsequent experiments. Previous studies have proven that glycolysis is one of the biological characteristics required for carcinogenesis and tumor progression in CRC [21-23]. To determine whether Tec inhibits the proliferation of HCT116 cells *via* regulating aerobic glycolysis, we performed Western blotting to detect the protein level of key glycolytic enzymes. The results showed that Tec downregulated the protein expression of PKM2, HK2, LDHA and GLUT1, which means that Tec is involved in blocking glycolysis in colorectal cancer cells Fig. (**1B**).

### 3.2 Tec Affected the Proliferation and Glycolysis of Colorectal Cancer Cells by Upregulating the Expression of miR-145

Studies have confirmed that mi-RNAs are widely involved in regulating various biological processes in CRC. To further investigate the molecular mechanisms of Tec-induced suppression of HCT116 cells proliferation and glycolysis, we focused on miR-145 and transfected miRNA-145 inhibitor or its corresponding control oligonucleotides (miR-NC) into HCT116 cells to explore the role miR-145 played in the pharmacologic effect of Tec. Our results showed that the expression of miR-145 was significantly elevated after Tec treatment for 24h Fig. (**2A**). Meanwhile, Fig. (**2B** and **C**) shows that viable HCT116 cells were increased when the expression of miR-145 was significantly decreased by miR-145 inhibitor. Moreover, glucose uptake and supernatant lactate levels in the Tec+ miR-145 inhibitor group significantly increased compared to those in the Tec+ miR-NC group Fig. (**2D** and **E**). Consistently, as shown in Fig. (**2F**), the protein expression of PKM2, HK2, LDHA and GLUT1 was further increased in the presence of Tec+ miR-145 inhibitor *versus* Tec+ miR-NC group. As per the above results, it can be concluded that Tec might inhibit proliferation and glycolysis *via* upregulating the expression of miR-145 in colorectal cancer cells.

### 3.3 Tec Inhibited the Proliferation and Glycolysis in Colorectal Cancer Cells *via* the lncRNA CCAT2/miR-145 Axis

Previous studies have verified that miR-145 could be regulated by lncRNA CCAT2 and lncRNA PVT1, which are associated with the malignant phenotype of several cancers [24, 25]. Therefore, the RT-qPCR technique was applied to investigate the expression of lncRNA CCAT2 and lncRNA PVT1 after Tec treatment for 24h. The results showed that compared to the untreated cells, the expression level of lncRNA CCAT2 dropped, while the lncRNA PVT1 expression seemed not to change significantly Fig. (**3A**). As shown in Fig. (**3B**), oe-CCAT2 significantly increased CCAT2 expression and significantly reduced miR-145 expression. To further ascertain the influence of lncRNA CCAT2 on proliferation in colorectal cancer cells, we monitored cell viability by CCK-8 assay. As shown in Fig. (**3C**), the upregulation of lncRNA CCAT2 expression increased cell viability. Next, we detected the level of glucose uptake and supernatant lactate after lncRNA CCAT2 overexpression. The results indicated that the upregulation of lncRNA CCAT2 caused the rise in glucose uptake and supernatant lactate level Fig. (**3D**). Similarly, western blotting results showed that the upregulation of lncRNA CCAT2 caused the rise in PKM2, HK2, LDHA, and GLUT1 protein expression levels Fig. (**3E**). Altogether, these results showed that Tec inhibited the proliferation and glycolysis *via* downregulating the expression of lncRNA CCAT2 in colorectal cancer cells.

**Fig. (3):**
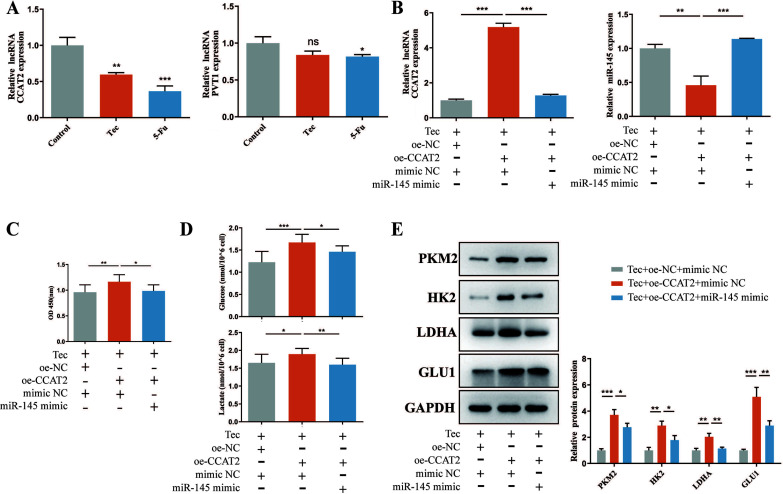
Tec inhibited the proliferation and glycolysis in colorectal cancer cells *via* the lncRNA CCAT2/miR-145 axis. (**A**) RT-qPCR was conducted to detect the expressions of lncRNA CCAT2 and lncRNA PVT1 in HCT116 cells after Tec or 5-Fu treatment. (**B**) RT-qPCR assay was utilized to detect the expressions of lncRNA CCAT2 and miR-145 in cells. (**C**) CCK-8 assay was employed to detect cell proliferation after transfection of oe-CCAT2 with or without miR-145 mimic. (**D**) The glucose uptake and supernatant lactate levels were detected by corresponding determination kits. (**E**) Western blot assay for HK2, LDHA, PKM2, and GLU1 was executed. *P<0.05, **P<0.01, ***P<0.001 *vs.* Control.

Furthermore, we co-transfected miR-145 mimic and oe-CCAT2 vector in Tec-treated HCT116 cells. RT-qPCR results showed that miR-145 mimic reversed the suppression of miR-145 expression that occurred in response to CCAT2 upregulation (Fig. **3B**). CCK-8 assay results showed that the miR-145 mimic reversed the cell proliferation increased by the oe-CCAT2 vector (Fig. **3C**). In addition, miR-145 mimic rescued the rise in glucose uptake, supernatant lactate level, and glycolysis-related protein expression levels caused by lncRNA CCAT2 overexpression (Fig. **3D**). Collectively, these results indicated that Tec inhibits the proliferation and glycolysis in colorectal cancer cells *via* regulating the lncRNA CCAT2/miR-145 axis.

### 3.4 Tec Inhibits the Growth of Colorectal Cancer *In vivo*

**Fig. (4):**
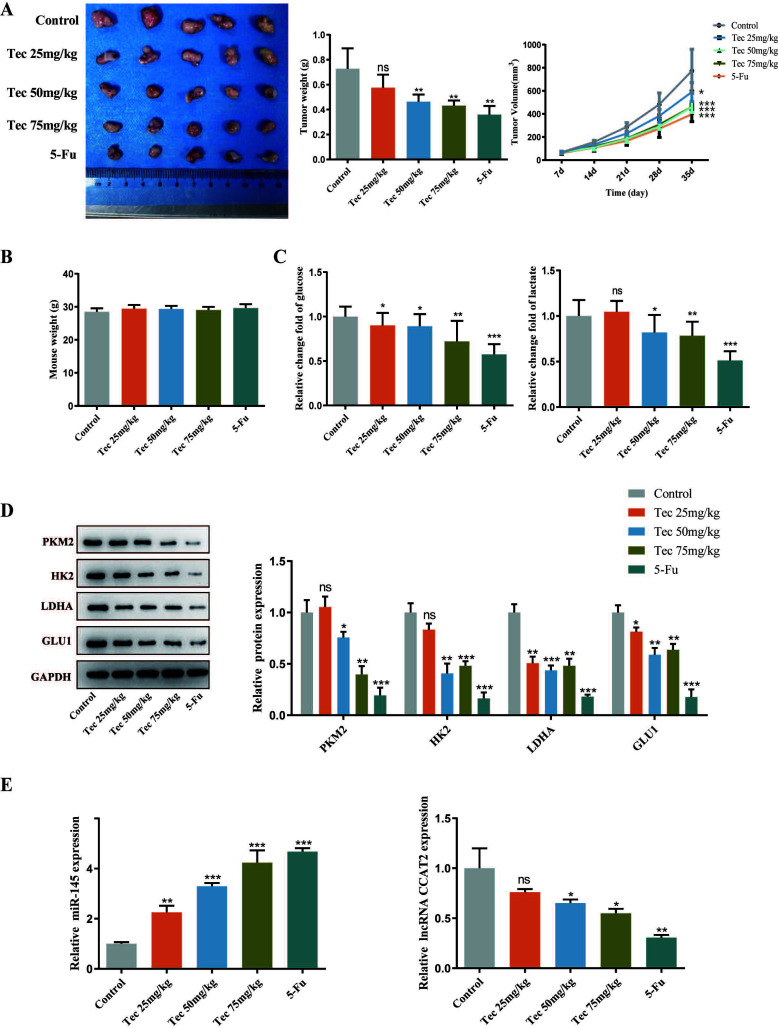
Tec inhibited the growth of colorectal cancer *in vivo*. (**A**) Images of the tumors and average weight of the tumors in each group following completion of treatment and tumor volume measured during the course of treatment. (**B**) Plot the body weight of tumor-bearing mice in each group. (**C**) The glucose uptake and lactate levels in plasma were detected. (**D**) Western blot revealed the expression of related protein expression in Tec-treated tumors. (**E**) Effect of Tec on the mRNA expression levels of lncRNA CCAT2 and miR-145 in tumour tissues. *P<0.05, **P<0.01, ***P<0.001 *vs.* Control.

To identify the anti-tumor effect of Tec *in vivo*, we generated a xenograft model bearing HCT116 cells using BALB/c nude mice. The volume and weight of tumors dose-dependently decreased in the Tec group compared to the control group. Besides, the antitumor effects of 50mg/kg Tec group and 75mg/kg Tec group were similar to those of 5-Fu group (Fig. **4A**). Notably, no significant difference in mice weight between the Tec groups and the control group was observed, and the mortality rate of the Tec groups was 0% (Fig. **4B**). Consistent with the findings *in vitro*, Tec significantly inhibited glycolysis *in vivo*, indicated by the decrease in glucose uptake and lactate level in plasma and related protein expression in Tec-treated tumors (Fig. **4C** and **D**). Finally, the expression levels of lncRNA CCAT2 and miR-145 were found to be decreased and increased by Tec treatment, respectively (Fig. **4E**). Altogether, these results emphasized that Tec inhibits tumor growth and glycolysis in CRC *via* mediating the lncRNA CCAT2/ miR-145 axis *in vivo*.

## 4 DISCUSSION

Colorectal cancer, as one of the most common prevalent malignancies, seriously threatens the healthy living of people due to its tumorigenesis, development, and metastasis [26]. Hence, identifying new drug targets and finding an effective and safe drug to treat colorectal cancer better are urgently needed. Recently, mounting attention has been paid to Chinese Traditional Medicine due to its therapeutic effects on tumors and its multi-target therapeutic mechanism. The novelty of the present study is to authenticate the effect of Tec on the proliferation and glycolysis of colorectal cancer *via* regulating the lncRNA CCAT2/ miR-145 axis *in vitro* and *in vivo*. Our findings identified a novel mechanism of Tec for inhibiting colorectal cancer progression and suggested the significant implication of lncRNA CCAT2/ miR-145 axis for the treatment of CRC.

Glycolysis is a metabolic process, in which cancer cells gain limited nutrients and acquire ATP and intermediate metabolites faster than other cells in the tumor microenvironment [27]. Glucose enters glycolysis-related pathways to produce fatty acids, amino acids, and nucleotides, which are fundamental to supporting cancer cell growth. The transformation of the glucose metabolism pattern leads to an increase in lactic acid and a decrease in pH in the microenvironment, resulting I n malignant progression [28, 29]. Evidence has shown that glycolysis is one of the critical anti-tumor targets, and inhibition of glycolysis can induce apoptosis, ferroptosis, and other types of programmed death in tumor cells [30-32]. During glycolysis, glucose enters the cell under GLUT mediation and is phosphorylated to G6P under the action of HK2 in the cytoplasm. Eventually, it was metabolized to pyruvate catalyzed by a variety of rate-limiting enzymes, such as PKM2 and LDHA [33]. Several natural anticancer compounds lead to glycolysis stagnation and thus become a hinder to cell growth [34-36]. In our results, it was revealed that Tec treatment could effectively inhibit the proliferation of HCT116 colorectal tumor cells, and the expression of PKM2, HK2, LDHA and GLUT1 protein decreased, suggesting that glycolysis might be the promising target of Tec for anti-colorectal cancer therapy.

Numerous studies have demonstrated that lncRNA/ miRNA crosstalk is closely related to glycolysis in various cancers. Su *et al.* [37] identified that the upregulated expression of lncRNA-LET could have reduced cell viability and inhibited glycolysis by targeting miR-93-5p in esophageal squamous cells. LncRNA LINC00261 was also reported to exert its biological function of blocking glycolysis by binding to miR-222-3p in pancreatic cancer [38]. LncRNA CCAT2, identified for the first time in 2013, was found to be highly expressed in CRC, and its abnormal expression can promote tumor growth, metastasis, and chromosome instability [39-41]. Recent research reported that colorectal cancer cell lines overexpressing lncRNA CCAT2 showed a higher level of glucose uptake, lactic acid secretion, and oxygen consumption [24]. Meanwhile, the mechanistic investigation revealed that the lncRNA CCAT2 regulates cancer metabolism *in vitro* and *in vivo* in an allele-specific manner by binding the Cleavage Factor I (CFIm) complex with distinct affinities for the two subunits (CFIm25 and CFIm68) [42]. Here, we first demonstrated a new molecular mechanism of Tec in promoting the expression of miR-145 to suppress glycolysis of colorectal cancer cells. Moreover, mechanism rescue experiments showed that co-transfection of lncRNA CCAT2 overexpressed vector with miR-145 mimics effectively reversed lncRNA CCAT2-induced glycolysis. At this point, we provide novel insight into lncRNA CCAT2/miR-145 axis regulated by Tec in the glycolysis of colorectal cancer.

It has been apparent that the regulatory mechanisms that exist between miRNAs and lncRNAs include miRNA-induced lncRNA decay, lncRNAs as miRNA sponges/decoys, lncRNAs competing with miRNAs for interaction with mRNAs, and lncRNAs generating miRNAs. Zhang *et al.* [43] found that CCAT2 could bind to miR‐145 and then inhibit the expression of miR‐145. Moradi *et al.* [44] also reported that there were potential binding areas between CCAT2 and hsa-miR-145-5p, and CCAT2 might regulate the activity of has-miR-145-5p according to bioinformatics analyses. Niu *et al.* [45] revealed that CCAT2 selectively blocked miR‐145 processing, leading to decreased mature miR‐145 presence. It is worth exploring the effect of Tec on the regulatory mechanisms between CCAT2 and miR-145 in our follow-up studies.

This study further supports the application evidence of Tec in cancer clinical treatment. However, we believe that the effect of Tec is more than this, and its anticancer mechanism based on other pathways and the range of safe applications still requires further investigation.

## CONCLUSION

In summary, we demonstrated that Tec inhibited cellular glycolysis-induced cell proliferation through the regulation of the lncRNA CCAT2/ miR-145 axis *in vivo* and *in vitro*. Our results not only provide new insight into the underlying mechanism of Tec-restrained glycolysis but also offer an essential clue for the development of native compounds to prevent progression in various cancers, including colorectal cancer.

## Figures and Tables

**Fig. (1):**
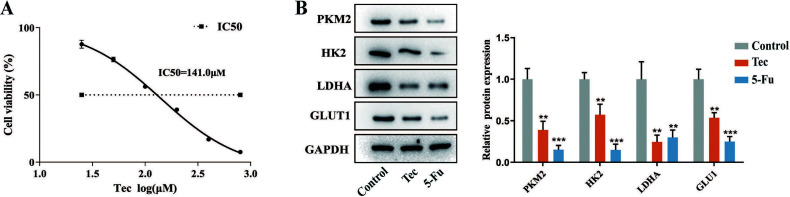
Tec suppressed the proliferation and glycolysis of colorectal cancer cells. (**A**) CCK-8 was used to detect the effect of Tec on cell viability in HCT116 cells. (**B**) Western blot was used to detect the expression of key glycolytic enzymes. **P<0.01, ***P<0.001 *vs.* Control.

**Fig. (2):**
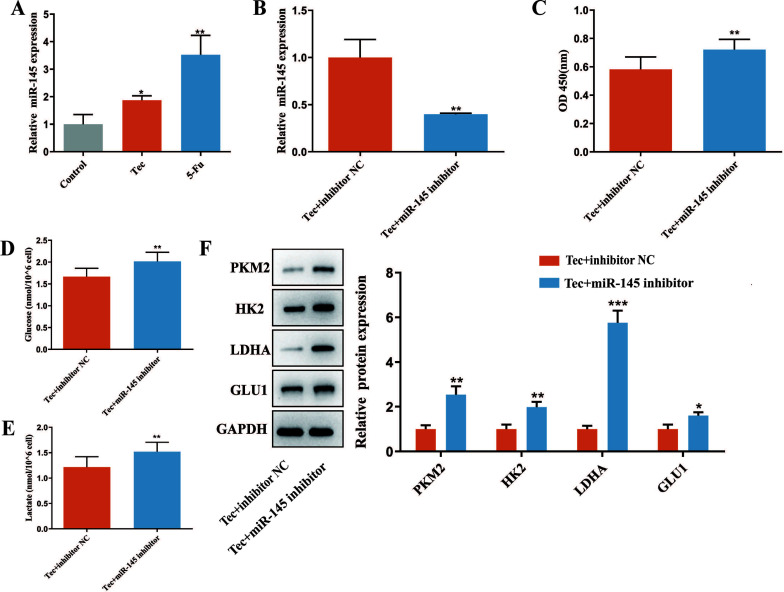
Tec affected the proliferation and glycolysis of colorectal cancer cells by upregulating the expression of miR-145. (**A**) RT-qPCR was used to detect the expression of miR-145 in HCT116 cells. (**B**) MiR-145 expression was detected by RT-qPCR after transfecting miRNA-145 inhibitor or miR-NC into HCT116 cells. (**C**) CCK-8 assay was employed to detect cell proliferation after the Tec treatment and transfection of miRNA-145 inhibitor. (**D** and **E**) The glucose uptake and supernatant lactate levels were detected by commercial assay kits. (**F**) Western blot was performed to detect the expressions of glycolysis-related proteins in cells treated with Tec and transfected with miRNA-145 inhibitor. *P<0.05, **P<0.01, ***P<0.001 *vs.* Control.

## Data Availability

The data and supportive information are available within the article.

## LIST OF ABBREVIATIONS

CRC: Colorectal Cancer
LncRNA: Long Non-coding RNA
CCK-8: Cell Counting Kit-8
RT-qPCR: Real Time Quantitative PCR
PKM2: M2-type Pyruvate Kinase
CCAT2: Colon Cancer-associated Transcript 2
MREs: miRNA Response Elements
FBS: Fetal Bovine Serum
RIPA: Radio Immunoprecipitation Assay
PMSF: Phenylmethanesulfonyl Fluoride
IC_50_: Half Maximal Inhibitory Concentration

## References

[1] Hossain M.S., Karuniawati H., Jairoun A.A., Urbi Z., Ooi D.J., John A., Lim Y.C., Kibria K.M.K., Mohiuddin A.K.M., Ming L.C., Goh K.W., Hadi M.A. (2022). Colorectal cancer: A review of carcinogenesis, global epidemiology, current challenges, risk factors, preventive and treatment strategies.. Cancers.

[2] Miller K.D., Nogueira L., Devasia T., Mariotto A.B., Yabroff K.R., Jemal A., Kramer J., Siegel R.L. (2022). Cancer treatment and survivorship statistics, 2022.. CA Cancer J. Clin..

[3] Holch J.W., Held S., Stintzing S., Fischer von Weikersthal L., Decker T., Kiani A., Kaiser F., Heintges T., Kahl C., Kullmann F., Scheithauer W., Moehler M., von Einem J.C., Michl M., Heinemann V. (2020). Relation of cetuximab-induced skin toxicity and early tumor shrinkage in metastatic colorectal cancer patients: results of the randomized phase 3 trial FIRE-3 (AIO KRK0306).. Ann. Oncol..

[4] Ye H., Wang K., Lu Q., Zhao J., Wang M., Kan Q., Zhang H., Wang Y., He Z., Sun J. (2020). Nanosponges of circulating tumor-derived exosomes for breast cancer metastasis inhibition.. Biomaterials.

[5] Ye H., Wang K., Zhao J., Lu Q., Wang M., Sun B., Shen Y., Liu H., Pané S., Chen X.Z., He Z., Sun J. (2023). In situ sprayed nanovaccine suppressing exosomal PD-L1 by golgi apparatus disorganization for postsurgical melanoma immunotherapy.. ACS Nano.

[6] Ortíz R., Quiñonero F., García-Pinel B., Fuel M., Mesas C., Cabeza L., Melguizo C., Prados J. (2021). Nanomedicine to overcome multidrug resistance mechanisms in colon and pancreatic cancer: Recent progress.. Cancers (Basel).

[7] Karthika C., Sureshkumar R., Zehravi M., Akter R., Ali F., Ramproshad S., Mondal B., Kundu M.K., Dey A., Rahman M.H., Antonescu A., Cavalu S. (2022). Multidrug resistance in cancer cells: Focus on a possible strategy plan to address colon carcinoma cells.. Life.

[8] Pavlova N.N., Zhu J., Thompson C.B. (2022). The hallmarks of cancer metabolism: Still emerging.. Cell Metab..

[9] Zhong X., He X., Wang Y., Hu Z., Huang H., Zhao S., Wei P., Li D. (2022). Warburg effect in colorectal cancer: The emerging roles in tumor microenvironment and therapeutic implications.. J. Hematol. Oncol..

[10] Chu Z., Huo N., Zhu X., Liu H., Cong R., Ma L. (2021). FOXO3A-induced LINC00926 suppresses breast tumor growth and metastasis through inhibition of PGK1-mediated Warburg effect.. Mol. Ther..

[11] Nava G.M., Madrigal Perez L.A. (2022). Metabolic profile of the Warburg effect as a tool for molecular prognosis and diagnosis of cancer.. Expert Rev. Mol. Diagn..

[12] Jing Z., Liu Q., He X., Jia Z., Xu Z., Yang B., Liu P. (2022). NCAPD3 enhances Warburg effect through c-myc and E2F1 and promotes the occurrence and progression of colorectal cancer.. J. Exp. Clin. Cancer Res..

[13] Lu T.X., Rothenberg M.E. (2018). MicroRNA.. J. Allergy Clin. Immunol..

[14] Takai T., Yoshikawa Y., Inamoto T., Minami K., Taniguchi K., Sugito N., Kuranaga Y., Shinohara H., Kumazaki M., Tsujino T., Takahara K., Ito Y., Akao Y., Azuma H. (2017). A novel combination RNAi toward warburg effect by replacement with miR-145 and silencing of PTBP1 induces apoptotic cell death in bladder cancer cells.. Int. J. Mol. Sci..

[15] Zhang S., Pei M., Li Z., Li H., Liu Y., Li J. (2018). Double‐negative feedback interaction between DNA methyltransferase 3A and microRNA‐145 in the Warburg effect of ovarian cancer cells.. Cancer Sci..

[16] Yu Y., Nangia-Makker P., Farhana L., Majumdar A.P.N. (2017). A novel mechanism of lncRNA and miRNA interaction: CCAT2 regulates miR-145 expression by suppressing its maturation process in colon cancer cells.. Mol. Cancer.

[17] Guo T., Li Y., Hong S., Cao Q., Chen H., Xu Y., Dai G., Shao G. (2022). Evidence for anticancer effects of chinese medicine monomers on colorectal cancer.. Chin. J. Integr. Med..

[18] Guo Y., Chen Y.H., Cheng Z.H., Ou-Yang H.N., Luo C., Guo Z.L. (2016). Tectorigenin inhibits osteosarcoma cell migration through downregulation of matrix metalloproteinases *in vitro*.. Anticancer Drugs.

[19] Amin A., Mokhdomi T.A., Bukhari S., Wani S.H., Wafai A.H., Lone G.N. (2015). Tectorigenin ablates the inflammation-induced epithelial-mesenchymal transition in a co-culture model of human lung carcinoma.. Pharmacol. Rep..

[20] Jiang C.P., Ding H., Shi D.H., Wang Y.R., Li E.G., Wu J.H. (2012). Pro-apoptotic effects of tectorigenin on human hepatocellular carcinoma HepG2 cells.. World J. Gastroenterol..

[21] Hu T., Liu H., Liang Z., Wang F., Zhou C., Zheng X., Zhang Y., Song Y., Hu J., He X., Xiao J., King R.J., Wu X., Lan P. (2020). Tumor-intrinsic CD47 signal regulates glycolysis and promotes colorectal cancer cell growth and metastasis.. Theranostics.

[22] Hong J., Guo F., Lu S.Y., Shen C., Ma D., Zhang X., Xie Y., Yan T., Yu T., Sun T., Qian Y., Zhong M., Chen J., Peng Y., Wang C., Zhou X., Liu J., Liu Q., Ma X., Chen Y.X., Chen H., Fang J.Y.F. (2021). nucleatum targets lncRNA ENO1-IT1 to promote glycolysis and oncogenesis in colorectal cancer.. Gut.

[23] Yu S., Zang W., Qiu Y., Liao L., Zheng X. (2022). Deubiquitinase OTUB2 exacerbates the progression of colorectal cancer by promoting PKM2 activity and glycolysis.. Oncogene.

[24] Pirlog R., Drula R., Nutu A., Calin G.A., Berindan-Neagoe I. (2021). The roles of the colon cancer associated transcript 2 (CCAT2) Long Non-Coding RNA in cancer: A comprehensive characterization of the tumorigenic and molecular functions.. Int. J. Mol. Sci..

[25] Shen S.N., Li K., Liu Y., Yang C.L., He C.Y., Wang H.R. (2020). RETRACTED: Silencing lncRNAs PVT1 upregulates mir-145 and confers inhibitory effects on viability, invasion, and migration in EC.. Mol. Ther. Nucleic Acids.

[26] Li J., Ma X., Chakravarti D., Shalapour S., DePinho R.A. (2021). Genetic and biological hallmarks of colorectal cancer.. Genes Dev..

[27] Vaupel P., Multhoff G. (2021). Revisiting the warburg effect: historical dogma versus current understanding.. J. Physiol..

[28] Lunt S.Y., Vander Heiden M.G. (2011). Aerobic glycolysis: Meeting the metabolic requirements of cell proliferation.. Annu. Rev. Cell Dev. Biol..

[29] Ghanavat M., Shahrouzian M., Deris Zayeri Z., Banihashemi S., Kazemi S.M., Saki N. (2021). Digging deeper through glucose metabolism and its regulators in cancer and metastasis.. Life Sci..

[30] Li M., Chen X., Wang X., Wei X., Wang D., Liu X., Xu L., Batu W., Li Y., Guo B., Zhang L. (2021). RSL3 enhances the antitumor effect of cisplatin on prostate cancer cells *via* causing glycolysis dysfunction.. Biochem. Pharmacol..

[31] Tan P., Li M., Liu Z., Li T., Zhao L., Fu W. (2022). Glycolysis-Related LINC02432/Hsa-miR-98-5p/HK2 axis inhibits ferroptosis and predicts immune infiltration, tumor mutation burden, and drug sensitivity in pancreatic adenocarcinoma.. Front. Pharmacol..

[32] Yao X., Li W., Fang D., Xiao C., Wu X., Li M. (2021). Emerging roles of energy metabolism in ferroptosis regulation of tumor cells.. Adv. Sci..

[33] Pan Y., Wang W., Huang S., Ni W., Wei Z., Cao Y., Yu S., Jia Q., Wu Y., Chai C., Zheng Q., Zhang L., Wang A., Sun Z., Huang S., Wang S., Chen W., Lu Y. (2019). Beta‐elemene inhibits breast cancer metastasis through blocking pyruvate kinase M2 dimerization and nuclear translocation.. J. Cell. Mol. Med..

[34] Li H., Hu S., Pang Y., Li M., Chen L., Liu F., Liu M., Wang Z., Cheng X. (2018). Bufalin inhibits glycolysis-induced cell growth and proliferation through the suppression of Integrin β2/FAK signaling pathway in ovarian cancer.. Am. J. Cancer Res..

[35] Hou J., Chen Q., Huang Y., Wu Z., Ma D. (2022). Caudatin blocks the proliferation, stemness and glycolysis of non-small cell lung cancer cells through the Raf/MEK/ERK pathway.. Pharm. Biol..

[36] Dai Y., Liu Y., Li J., Jin M., Yang H., Huang G. (2022). Shikonin inhibited glycolysis and sensitized cisplatin treatment in non-small cell lung cancer cells *via* the exosomal pyruvate kinase M2 pathway.. Bioengineered.

[37] Su X., Xue C., Xie C., Si X., Xu J., Huang W., Huang Z., Lin J., Chen Z. (2022). lncRNA-LET regulates glycolysis and glutamine decomposition of esophageal squamous cell carcinoma through miR-93-5p/miR-106b-5p/SOCS4.. Front. Oncol..

[38] Zhai S., Xu Z., Xie J., Zhang J., Wang X., Peng C., Li H., Chen H., Shen B., Deng X. (2021). Epigenetic silencing of LncRNA LINC00261 promotes c-myc-mediated aerobic glycolysis by regulating miR-222-3p/HIPK2/ERK axis and sequestering IGF2BP1.. Oncogene.

[39] Xin Y., Li Z., Zheng H., Chan M.T.V., Ka Kei Wu W. (2017). CCAT 2: A novel oncogenic long non‐coding RNA in human cancers.. Cell Prolif..

[40] Chen B., Dragomir M.P., Fabris L., Bayraktar R., Knutsen E., Liu X., Tang C., Li Y., Shimura T., Ivkovic T.C., Cruz De los Santos M., Anfossi S., Shimizu M., Shah M.Y., Ling H., Shen P., Multani A.S., Pardini B., Burks J.K., Katayama H., Reineke L.C., Huo L., Syed M., Song S., Ferracin M., Oki E., Fromm B., Ivan C., Bhuvaneshwar K., Gusev Y., Mimori K., Menter D., Sen S., Matsuyama T., Uetake H., Vasilescu C., Kopetz S., Parker-Thornburg J., Taguchi A., Hanash S.M., Girnita L., Slaby O., Goel A., Varani G., Gagea M., Li C., Ajani J.A., Calin G.A. (2020). The Long Noncoding RNA CCAT2 induces chromosomal instability through BOP1-AURKB Signaling.. Gastroenterology.

[41] Wang D., Li Z., Yin H. (2021). Long Non-Coding RNA CCAT2 activates rab14 and acts as an oncogene in colorectal cancer.. Front. Oncol..

[42] Redis R.S., Vela L.E., Lu W., Ferreira de Oliveira J., Ivan C., Rodriguez-Aguayo C., Adamoski D., Pasculli B., Taguchi A., Chen Y., Fernandez A.F., Valledor L., Van Roosbroeck K., Chang S., Shah M., Kinnebrew G., Han L., Atlasi Y., Cheung L.H., Huang G.Y., Monroig P., Ramirez M.S., Catela Ivkovic T., Van L., Ling H., Gafà R., Kapitanovic S., Lanza G., Bankson J.A., Huang P., Lai S.Y., Bast R.C., Rosenblum M.G., Radovich M., Ivan M., Bartholomeusz G., Liang H., Fraga M.F., Widger W.R., Hanash S., Berindan-Neagoe I., Lopez-Berestein G., Ambrosio A.L.B., Gomes Dias S.M., Calin G.A. (2016). Allele-specific reprogramming of cancer metabolism by the long non-coding RNA CCAT2.. Mol. Cell.

[43] Zhang Z., Wang X., Wang Y., Zhou D., Wu H., Cheng W., Wang Q., Zheng G., Wang J., Gu J. (2022). Effect of long noncoding RNA CCAT2 on drug sensitivity to 5‐fluorouracil of breast cancer cells through microRNA‐145 meditated by p53.. J. Biochem. Mol. Toxicol..

[44] Moradi F, Mohajerani F, Sadeghizadeh M (2022). CCAT2 knockdown inhibits cell growth, and migration and promotes apoptosis through regulating the hsa-mir-145-5p/AKT3/mTOR axis in tamoxifenresistant MCF7 cells.. Life Sci.

[45] Niu C., Wang L., Ye W., Guo S., Bao X., Wang Y., Xia Z., Chen R., Liu C., Lin X., Huang X. (2020). CCAT2 contributes to hepatocellular carcinoma progression *via* inhibiting miR‐145 maturation to induce MDM2 expression.. J. Cell. Physiol..

